# Changing Strategies for the Detection of Bacteria in Platelet Components in Ireland: From Primary and Secondary Culture (2010–2020) to Large Volume Delayed Sampling (2020–2023)

**DOI:** 10.3390/microorganisms11112765

**Published:** 2023-11-14

**Authors:** Niamh O’Flaherty, Louise Bryce, James Nolan, Mark Lambert

**Affiliations:** Irish Blood Transfusion Service, National Blood Centre, D08 NH5R Dublin, Ireland; louise.bryce@ibts.ie (L.B.); mark.lambert@ibts.ie (M.L.)

**Keywords:** bacteria, contamination rates, platelet components (PCs), bacterial culture, risk, transfusion, safety

## Abstract

Bacterial contamination of platelet components (PC) poses the greatest microbial risk to recipients, as bacteria can multiply over the course of PC storage at room temperature. Between 2010 and 2020, the Irish Blood Transfusion Service (IBTS) screened over 170,000 buffy coat-derived pooled (BCDP) and single-donor apheresis platelets (SDAPs) with the BACT/ALERT 3D microbial detection system (Biomerieux, L’Etoile, France), using a two-step screening protocol which incorporated primary and secondary cultures. Although the protocol was successful in averting septic transfusion reactions (STRs), testing large sample volumes at later time points was reported to improve detection of bacterial contamination. A modified large-volume delayed sampling (LVDS)-type protocol was adopted in 2020, which in the case of SDAP was applied to collections rather than individual splits (2020–2023, 44,642 PC screened). Rates of bacterial contamination for BCDP were 0.125% on Day-2, 0.043% on Day-4 vs. 0.191% in the post-LVDS period. SDAP contamination rates in the pre-LVDS period were 0.065% on Day-1, 0.017% on Day-4 vs. 0.072% in the post-LVDS period. Confirmed STRs were absent, and the interdiction rate for possibly contaminated SDAP was over 70%. In the post-LVDS period, BCDPs had a higher total positivity rate than SDAPs, 0.191% (1:525) versus 0.072% (1:1385), respectively, (chi-squared 12.124, 1 df, *p* = 0.0005). The majority of organisms detected were skin-flora-type, low pathogenicity organisms, including coagulase-negative staphylococci and *Cutibacterium acnes*, with little change in the frequency of clinically significant organisms identified over time. Both protocols prevented the issue of potentially harmful components contaminated (rarely) with a range of pathogenic bacteria, including *Escherichia coli, Serratia marcesens*, *Staphylococcus aureus*, and streptococci. Culture positivity of outdates post-LVDS whereby 100% of expired platelets are retested provides a residual risk estimate of 0.06% (95% CI 0.016–0.150). However, bacterial contamination rates in expired platelets did not demonstrate a statistically significant difference between the pre-LVDS 0.100% (CI 0.033–0.234) and post-LVDS 0.059% (0.016–0.150) periods (chi-squared = 0.651, 1 df, *p* = 0.42).

## 1. Introduction

In a 1959 editorial on the ‘*precautions taken against’* bacterial contamination of blood products, a subject of the 7th ISBT Conference in Rome of that year, JD James wrote ‘*a mere stroke of cruel chance may lead to tragedy’* [1]. This statement still rings true today as transfusion-associated bacterial sepsis (TABS) remains a serious issue for blood services internationally.

The measures in variable use and/or recommended at that time in Europe included single-use sterilised equipment and collection bottles, skin disinfection with ether soap followed by 70% alcohol with chlorhexidine, avoidance of blood bottle manipulation after collection and processing, refrigeration of whole blood at 4 °C, inspection for signs of gross contamination, and investigation and reporting of contamination events, all of which led to enhanced safety of blood transfusion at the time [1,2]. The ensuing years after James’s paper, however, demanded a focus on viral safety, owing to the urgent need for serological and nucleic acid blood-donor screening tests to address the alarming number of transfusion transmitted infections (TTIs) caused by Hepatitis A, Hepatitis B, Hepatitis C viruses, and later HIV. During this time, the issue of bacterial contamination was largely disregarded, and little further progress was made. A rise in incidence of fatal septic transfusion reactions (STRs) notified through mandatory reporting channels brought bacterial safety back to the fore in the 1980s and 1990s, particularly for room-temperature-stored platelet components, by now in use for 50 years [3,4].

Further improvements in bacterial safety involved the use of plastic polymer sterile collection packs, closed systems, accessed if required with the use of sterile connection devices. In addition, changes to donor selection criteria regarding recent gastrointestinal illness contributed to a reduction in *Yersinia* infections in recipients, a relatively frequent and sometimes fatal transfusion transmitted bacterial infection (TTBI) in the 1980s and 1990s [5].

As unlike viruses, most bacteria can multiply over the course of the room-temperature-stored platelet concentrates’ shelf life, storage time limitations varying from 72 h to 7 days is perhaps the most variable and debated safety measure [6]. Many services use their own scientific and anecdotal data, coupled with local user supply requirements to inform storage times that are both safe and practical.

Skin-surface derived Gram-positive organisms comprise the most frequently implicated group of bacteria in platelet contamination. Unlike blood-borne viruses (BBVs), these microorganisms are part of the skin microbiome of blood donors worldwide; providing a protective barrier against colonisation of other agents, but harmful when transfused in a large volume or high concentration to vulnerable recipients. Improved skin disinfection protocols, emphasizing ‘no retouch’ techniques and importantly the diversion of the initial blood volume obtained from the donor were popularised in the 2000s and offered at least a 70% reduction in contamination levels [7].

However, bacteria still enter blood and platelet collection bags at rates of 1/1000–2500, well in excess of transfusion relevant viruses. This can come as a surprise as the procedures for blood-donor skin disinfection were informed by those used for ‘invasive’ healthcare-associated procedures requiring an aseptic environment. Gram-negative contaminations, which can cause the rapid demise of a recipient, are not averted by skin cleansing. Indeed the introduction of primary culture in the late 1990s and 2000s was key to reducing the risk of fatal STRs [8].

Since 2018, seven platelet STRs, some of which were fatal, have been reported in the United States, involving closely related isolates of *Acinetobacter *spp., *Staphylococcus saprophyticus*, and *Leclercia adecarboxylata* with a probable common source. This cluster of cases has been a cause of concern, as bacterial risk-reduction measures were applied in the majority of cases [9,10,11,12].

From the mid-2000s onwards and by 2009 the IBTS had introduced risk-reduction measures targeting platelet bacterial contamination with bacterial culture, volume diversion, and improved skin disinfection. Pressure remains on blood services to evaluate new strategies to mitigate bacterial risk for platelet components (PCs), with many opting for large-volume delayed sampling (LVDS) protocols to enhance bacterial detection. The subject of this report is the description of our findings from 10 years of a primary and secondary culture protocol (2010–2020) followed by almost 3 years of an LVDS-type screening protocol.

## 2. Materials and Methods

### 2.1. Platelet Products at the IBTS

The IBTS produces buffy coat-derived platelet pools (BCDP) and single-donor apheresis platelets (SDAP).

Platelet pools are prepared using the TACSI^®^ PL System for Platelet Pool preparation (TerumoBCT, Lakewood, CO, USA), after an overnight hold of the whole blood unit at 22 °C. Four buffy coats from individual donors are pooled with approximately 25 mL of plasma from each donor, and resuspended in approximately 70% Platelet Additive Solution (PAS-E; Macopharma SSP+^TM^).

SDAP are collected using Trima Accel^®^ Automated Blood Collection System (TerumoBCT, Lakewood, CO, USA) and suspended in 100% plasma. Approximately 77.5% of SDAP collections yield triple doses (TDs), 17.4% are double doses (DDs) and 5.1% yield a single dose (SD) of platelets. The proportion of all platelets collected by apheresis is approximately 60–70%; prior to 2019, the ratio of SDAP to BCDP was 80:20. All platelets are leucodepleted as part of the manufacturing process. (See Table S6, and Section A).

In the Supplementary Material (Section A), we included details of Bioburden Reduction, Donor Follow-Up Associated with Positive BACT/ALERT Bottle, and Management of Patients Transfused with Platelets Linked to a Positive BACT/ALERT Bottle.

### 2.2. Bacterial Screening of Platelets

#### 2.2.1. Pre-November 2020 (Pre-LVDS)

In late 2004, the IBTS commenced screening of all platelet products for the presence of bacteria using the BACT/ALERT 3D Blood Culture System (BioMérieux, Durham, NC, USA) [13] SDAP collections were sampled on Day-1, following a period of ≥12 h post-phlebotomy; BCDP were sampled on Day-2, following a period of ≥12 post-*manufacture* (≥36 h from phlebotomy). By the end of 2005, secondary culture was performed on Day-4, which allowed extension of expiry to Day-7; this involved *individual* sampling of SDAP splits. Sample volume was 16 mL in total, BPA and BPN bottles each being inoculated with 8 mL of platelet product, as previously described [13]. Once incubation had commenced, platelet products were immediately available for issue, as ‘negative-to-date’ (assuming all other mandatory testing was completed). See Table 1 for details of platelet component management.

#### 2.2.2. Post-November 2020 (Post-LVDS)

In late 2020, the IBTS adopted a modified * large-volume delayed sampling (LVDS) single-test protocol for all platelet components. The sample for bacterial screening, obtained on Day-2, must be delayed until ≥36 h post-phlebotomy for both BCDP and SDAP. An 8 mL sample is inoculated into each of a BPA and BPN bottle from the BCDP or the ‘mother-bag’ (again 16 mL in total being sampled), rather than the individual splits of SDAP [14,15]. A 12 h hold after bottle loading was also introduced at this time. All platelet products at the IBTS now have a 7-day expiry; BPA and BPN bottles are incubated until Day-8.

#### 2.2.3. Testing at Expiry

As described by Murphy et al., expired units are retested with a 10 mL sample inoculated into an aerobic and anaerobic bottle on BACT/ALERT 3D and incubated for 7 days [13]. Positive flags from the BACT/ALERT 3D instrument are investigated as described below with the initial positive bottle referred to the reference laboratory for Gram stain, culture, and identification. Full investigation of associated unissued components (e.g., RCC, plasma, associated SDAP splits) is also undertaken, including reincubation of the unit. In the pre-LVDS period, only a small proportion of all platelets remaining at expiry were retested. In the post-LVDS period, all unissued platelet components have been tested at expiry for bacteria missed by live, in-date testing.

### 2.3. Processing of Positive BACT/ALERT Bottles

Positive flags on the BACT/ALERT 3D instrument trigger an alarm on our environmental monitoring system (REES Scientific, Trenton, NJ, USA); this is achieved through a voltage change at the monitoring probe. IBTS scientists are then alerted by telephone in relation to the positive flag (24/7/365). The positive BACT/ALERT 3D bottles are removed and referred to the medical microbiology laboratory of a large teaching hospital on the shared campus of our blood centre in Dublin. Bottles are subcultured by the IBTS onto Columbia Blood and Chocolate Blood agar under aerobic and anaerobic conditions (30–35 °C) and read after a minimum of 48 h. A Gram stain is either performed by scientists in the IBTS or, alternatively, a Gram stain is requested of and then reported by the reference laboratory ‘urgently‘, if the PC or co-components have already been infused. Full identification of cultured isolates is performed using MALDI-TOF methodology (Vitek MS; BioMérieux, Durham, NC, USA); an antimicrobial sensitivity profile is provided when required.

### 2.4. Product Recall

Product recall is initiated immediately for all relevant products, which in the case of a platelet pool, includes four associated red cells and plasma components (the vast majority of plasma produced by the IBTS is not currently for clinical use). All recalled products are investigated in the same manner as the primary culture. Medical teams are informed of the initial Gram stain and final culture result of contaminated components; we record the status of the recipient and their outcome.

Recalled platelets are examined for signs of discolouration, aggregates, and leaks prior to testing. A 10 mL sample is inoculated into both BPA and BPN bottles and incubated for 7 days. The platelet/blood packs are then incubated at 32–35 °C for 3 days and tested again for an additional 7 days.

### 2.5. Classification of Positive Platelets

Classification of Positivity is dependent on ability to confirm the presence of bacteria in associated products [8,13].

- Confirmed Positive refers to a positive signal on BACT/ALERT 3D, a positive subculture from the BACT/ALERT bottle, and a further culture of the same species from the platelet unit, or, for pools, from the pool or from one other component from the donations used in the pool.
- False Positive refers to a positive signal on BACT/ALERT 3D, but no organism detected on subculture or Gram stain of the associated bottles, or on reculture of the unit.
- Indeterminate Positive (adapted from Benjamin et al. [8])*:* positive subculture from the initial BACT/ALERT bottle but not confirmed on subsequent reculture of remaining components, irrespective of the proportion of the associated components available for testing (i.e., all components may have been available for testing *, or only a proportion of them); or no residual components were available for retesting. * Please note these cases are not considered false positive by our definitions.

## 3. Results

The primary culture obtained from SDAP and BCDP are reported under ’Day-1 or -2 testing‘ (day of donation being Day-0). A proportion of SDAP and BCDP underwent a second test approximately 4 days post-donation and are referred to here as Day-4 testing. The platelets screened from 2010 to 5 November 2020 are referred to as ‘pre-LVDS’ and the platelets screened from 6 November 2020 to June 2023 are referred to as ‘post-LVDS’.

### 3.1. Pre-LVDS Period

Pre-LVDS, 175,229 platelets were screened on Day-1 or Day-2 post-donation, with a further 112,469 tests being performed at Day-4. Of the 287,698 tests conducted on platelets, 178 flagged with positive cultures (0.062%; see Table 2 and Table 3); 101 were in the confirmed positive category (0.035%) and 77 were in the indeterminate category. SDAPs had a positivity rate of 0.065% (confirmed 0.026% and indeterminate 0.039%) on Day-1 and 0.017% on Day-4 (note the rate is representative of multiples of apheresis splits for which it is not possible to account for retrospectively and may indicate an artificially low rate; confirmed 0.009% and indeterminate 0.008%). Samples from SDAP yielded 48% of the total positive cultures for the period. BCDP screened on Day-2 had a positivity rate of 0.125% (confirmed 0.088% and indeterminate 0.037%) and 0.043% on Day-4 (confirmed 0.030% and indeterminate 0.013%) and accounted for 52% of all culture positive samples pre-November 2020.

Two hundred and two BACT/ALERT 3D machine signals were categorised as false positive in the 10 years pre-LVDS, with a rate of 0.070% (1:1424). SDAP had lower rates of false positives on both Day-1 (0.086% (1:1163)) and Day-4 (0.032% (1:3083)) compared to BCDP samples, which generated false positive rates of 0.104% (1:966) and 0.048% (1:2095) on Day-1 and Day-4, respectively.

### 3.2. Post-LVDS Period

The positivity rate for all platelets in the post-LVDS period was 0.128%, with 0.090% (1:1116) confirmed and 0.038% (1:2626) indeterminate (see Table 3 and Table 4). BCDPs had a higher total positivity rate than SDAPs, 0.191% (1:525) versus 0.072% (1:1385), respectively (chi-squared 12.124, 1 df, *p* = 0.0005) Only three samples (0.007%) were categorised as false positive post-LVDS. These false flags were generated from SDAPs 0.013% (1:7846).

### 3.3. Organisms Isolated Pre-LVDS

**Figure 1:**
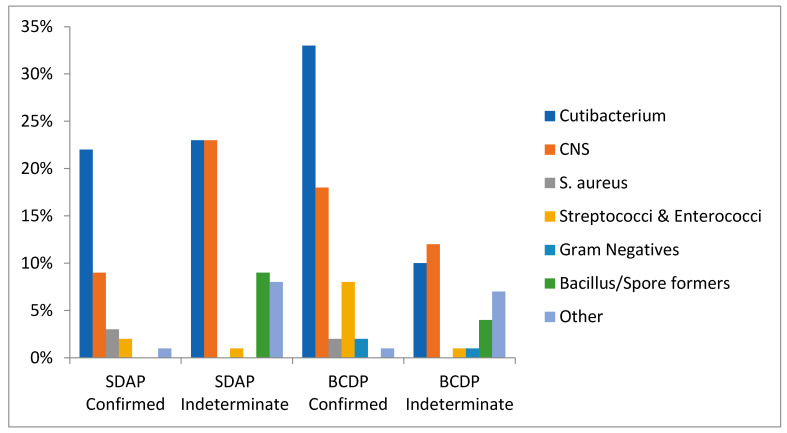
Frequency of organisms detected as a percentage of all confirmed or indeterminate investigations for each platelet type pre-LVDS.

Pre-LVDS, 101 organisms were identified in confirmed investigations out of 287,698 platelets tested on Day-1, -2, -4 (see Table 2, Table 5 and Table S3; Figure 1). Of the 178 positive cultures from confirmed and indeterminate cases, 80 (45%) were infused prior to recall (Table 2(T2) and Table S1). Skin commensals were the most commonly identified group of organisms; *Cutibacterium acnes* (previously *Propionibacterium acnes*) was isolated in 54% (n = 55) and coagulase-negative staphylococci (CNS) in 27% (n = 27) of confirmed investigations. The indeterminate category yielded a similar profile, with *Cutibacterium* spp. and CNS being identified in 33% (n = 26) and 35% (n = 27) of positive cultures, respectively (Table S1). *Staphylococcus aureus* was isolated in five (5%) confirmed cases but evaded the screening test twice thus indicating a false negative test. The units were recalled for reculture based on abnormalities observed on visual inspection. Gram-negative organisms were identified in only two (2%) confirmed (*E. coli* and *S. marcesens*, interdicted) and three (4%) indeterminate cases in a 10-year period (pre-LVDS). The organisms isolated in the indeterminate cases were likely to be contaminants (*Bacteroides, Desulfovibrio, Acinetobacter*) as they failed to culture from the resampled residual components.

### 3.4. Co-Components

In the 10-years pre-LVDS, there were 91 positive cultures derived from BCDP samples. Of these, 47% were associated with a component infused prior to recall. In 41%, the pool alone was infused, in 2% the red cell was also infused, and in 4% the red cell only was infused. In 87 positive SDAPs, 43% were infused. In 28%, all splits were infused and in 15% an associated split had been infused at the time of recall (see Table 6). For 42 of the positive SDAP culture results, multiple splits from a donation were available for confirmatory testing. In 73% (31/42), more than one split was contaminated with the same organism (pre-LVDS confirmed data). For BCDP, red cell components were contaminated with the same organism as the pool sample in 29.8% of the cases (where the component was available for testing).

### 3.5. Organisms Isolated Post-LVDS

In the three years since the change to an LVDS type protocol and 12 h hold, we recorded 43 confirmed and 18 indeterminate positives out of 51,451 platelets tested on Day-2 or at outdate (see Table 7, Table 8, and Table S2; Figure 1). This includes 30 confirmed and 11 indeterminate BCDPs and 13 confirmed and 7 indeterminate SDAPs. Skin commensals continue to be the most abundant group of organisms isolated. *Cutibacterium acnes* accounted for 56% (n = 24) and 61% (n = 11) of the organisms found in confirmed and indeterminate cases, respectively. Coagulase-negative staphylococci were found in 28% of both confirmed (n = 12) and indeterminate (n = 5) cases. Nearly all CNS organisms flagged within the first 10 h of monitoring on the BACT/ALERT 3D except for *Staphylococcus saccharolyticus*, which had a mean TTD of approximately 50 h.

Of the 61 confirmed and indeterminate (43 and 18) positive detections for which 25 (41%) were already infused, *C. acnes* accounted for 93% (n = 13, confirmed) and 82% (n = 9, indeterminate), respectively. *C. acnes* is also the organism that had the longest time to detection, 100 h on average. *S. aureus* was isolated in four of the confirmed cases (none infused), one of those, an expired SDAP, was missed on the first test. Further information on *S. aureus* contamination of PC can be found in the data reported in Supplementary Materials. No Gram-negative organisms have been detected since the introduction of LVDS.

Of the 40 positive BCDP, 53% were infused at the time of detection. In 25%, the pool only was infused, in 20% the red cell was also infused, and in 8% the red cell *only* was infused. Of the 17 positive apheresis platelets, 24% were infused. In 18% all splits had been infused and 6% had an associated split infused prior to recall (see Table 6).

### 3.6. Positivity According to BACT/ALERT Bottle Type BPA/BPN

**Figure 2:**
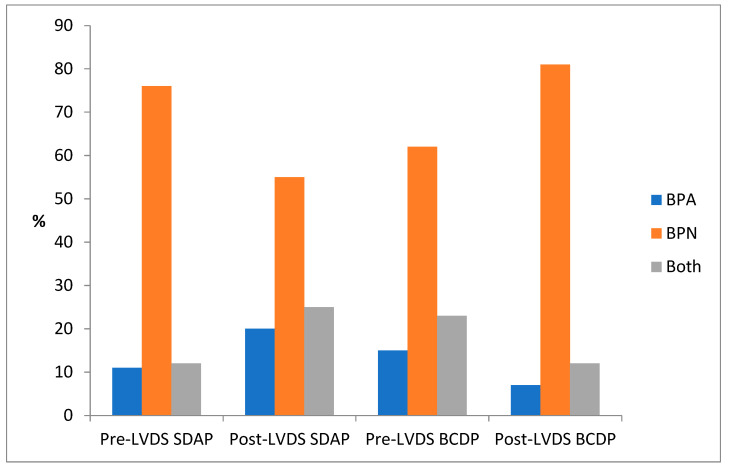
Frequency of confirmed and indeterminate positive cultures per bottle type.

The anaerobic bottle yielded the greatest proportion of contaminating organisms in all platelet components (average 68% pre- and post-LVDS). Whilst the majority of organisms in SDAP continued to be isolated from BPN bottles post-LVDS, there was a notable decrease from 76% to 55% in the proportion of anaerobic bottle detections. This change was coupled with an increase in the rate of positive detections observed in both bottles, and the aerobic bottle alone (12 vs. 25% and 11 vs. 20%, respectively) (see Figure 2). In contrast, there was an increase in BCDP positivity in BPN bottles (anaerobic) 62 vs. 81% pre- and post-LVDS, with a corresponding decrease in single bottle aerobic (15 vs. 7%) and double bottle positivity (23 vs. 12%). This observation ties in with the increased detection of *S. saccharolyticus* and *C. acnes* in the post-LVDS timeframe.

### 3.7. Positivity Rates over Time

**Figure 3:**
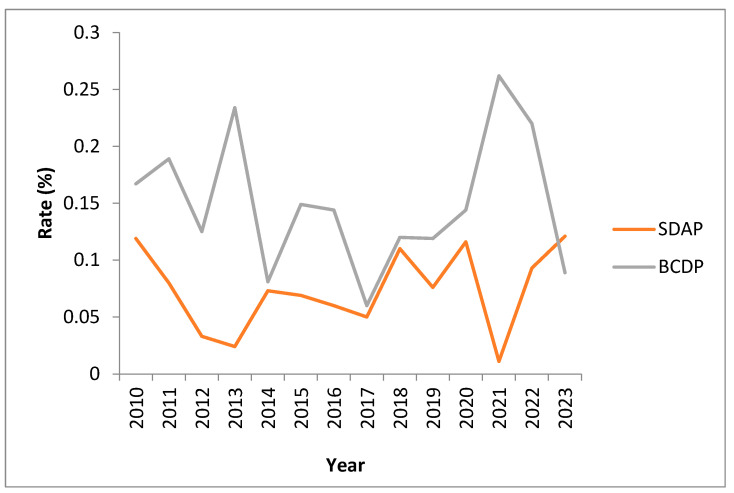
Combined positivity rates for SDAP and BCDP platelets over time.

Combined positivity rates for BCDP and SDAP range between 0.06% and 0.26% for BCDP (average 0.15%) and 0.01% and 0.12% for SDAP (average 0.07%). (See Figure 3). Rates are monitored monthly and significant increases have been investigated over the years with no clear identifiable root cause. Anecdotal evidence has directed us towards a potential association between increased rates of contamination with skin-flora-type organisms during the summer (warmer) periods.

### 3.8. Residual Risk: Outdate Testing and False Negative BACT/ALERT Screening Results

In the ten years pre-LVDS, a total of 4987 PCs that remained at expiry were retested at Day-5 or -7. This represented three percent of all platelet collections for the period (4987/175,229). The overall positivity rate was 0.100% (CI 0.033–0.234), representing n = 5 positives. The SDAP rate of 0.085% (3/3519, CI 0.018–0.249) was not significantly different to the BCDP rate of 0.136% (2/1468, CI 0.017–0.491); (chi-square = 0.2683, 1 df, *p* = 0.604487) (see Table 9). One out of the three positives in expired SDAP were deemed a probable contaminant as both splits were available for culture and tested negative (*Clostridium perfringens*). In the other cases, at least one split was already infused, but the other available split(s) was retested and negative (*Corynebacterium* spp. ×2). For BCDP, there was one confirmed *S. epidermidis* contamination at expiry, cultured from plasma and four RCCs, and one indeterminate positive, a probable contaminant with *Bacillus* spp. where all available material tested negative on reculture (see Table S5, Table 3).

Since the transition to LVDS in November 2020, 100% of platelets are screened upon expiry (Day-8) to establish the rate of false negative results. Four positive cultures (0.059%) were obtained from screening a total of 6809 expired platelets (5540 SDAP, 1269 BCDP) (three confirmed (two in SDAP, *Cutibacterium* spp. ×1, *S. aureus* ×1; one in BCDP *Cutibacterium* sp.)) and one indeterminate positive in SDAP with *Cutibacterium* sp. (where the remaining components tested negative) (see Table 8 and Table S5). In addition, *S. aureus* was isolated from an expired SDAP after aggregates were noted in both splits. The overall rates of detection in expired platelets did not demonstrate statistically significant difference in the pre-LVDS 0.100% (CI 0.033–0.234) and post-LVDS 0.059% (0.016–0.150) periods (chi-squared = 0.651, 1 df, *p* = 0.42). Further information on expired platelets and false negatives is contained in Table S5 and Section A7.

## 4. Discussion

According to the European Commission, between 2010 and 2016, 1% of notifications of serious adverse reactions in recipients concerned transfusion-transmitted infections (TTIs); 63% of these infections were caused by bacterial contamination [16]. Nonfatal and fatal STRs are estimated to occur with every 100,000 and 0.5–1 million platelet components transfused, respectively. Clinically unapparent bacterial contamination of transfused PCs is much more common, with a residual risk of approximately 1 in 10,000 [17].

As in many other countries, Ireland’s commitment to blood safety is evidenced in IBTS modelling calculations, which estimate a residual risk for BBV of less than 1 in 5 million donations. In contrast to virus-related risk, the continuous and incremental improvements in bacterial safety described earlier, have not led to as dramatic a reduction in the rate of bacterially contaminated PC. This is a gap that blood operators globally have a challenge in narrowing, even with screening-independent methods like pathogen-reduction technology [18,19].

### 4.1. IBTS Data on Bacterial Contamination of Platelets 2010–2020

A review of local and international data and bacterial mitigation procedures in 2018 and 2019 highlighted the limitations of an early sampling protocol and therefore an opportunity to reduce the potential for false-negative ‘early’ bacterial screens was identified [14,20,21]. Extending the bacterial sampling interval from a minimum of 12 to 36 h for apheresis donations was an achievable and logical goal. In addition, the interdiction of a *Serratia marcesens*-contaminated BCDP after 6 h of incubation prompted the evaluation and ultimately implementation of a single test, LVDS-type strategy and a 12 h quarantine/minimum incubation period. These two measures involved significant operational and logistical challenges for the organisation. The marked 90% reduction in STRs, achieved by the NHSBT with an LVDS approach, provided compelling evidence in support of change [14]. Equally encouraging were Canadian Blood Service (CBS) data that demonstrated success using a similar methodology for reducing STRs by a factor of three, as reported by Ramirez-Arcos et al. [15]. Additional advantages of proceeding with a change to single test-LVDS included a consistent approach to screening all platelets, as well as improved inventory management and a reduction in wastage and outdates. The reduction in wastage and outdates from an average 12.9% pre-LVDS, 9.4% in 2021, and 8.6% in 2022, has been one of the most beneficial and tangible benefits of streamlining platelet availability (B. Doyle. (IBTS, NBC, Dublin, Ireland). Personal communication, 2023) [22].

### 4.2. Buffy Coat-Derived Platelet data—Cessation of the Second Day-4 Test

In contrast to SDAP donations, the sampling interval was already ≥36 h post-collection for the BCDPs, so a conceivable disadvantage of the ‘single-test/7-day expiry’ protocol was the cessation of the Day-4 test (second bacterial culture), which at the time was conducted on approximately 30% of residual BCDP inventory. Analysis of the available data for the risk assessment of changing to a single-test protocol demonstrated the combined rate at which bacteria were identified from the primary and secondary screen of pooled platelets for the 10-year period (over 80,000 BCDP tests) was >0.10% (see Table 2). The value of the second Day-4 test was observed in the ‘slip through’ rate of 0.043%. Second test culture positives were infrequent and yielded pathogenic organisms on just two occasions (*S. aureus* ×1 (interdicted), one microaerophillic *Streptococcus* (infused)). Sepsis did not occur with un-intercepted, confirmed (or indeterminate) secondary culture positive BCDP components, which yielded skin-flora-type organisms in the majority (see Table 5). The second Day-4 test was somewhat undervalued by the lack of quarantine and its retrospective nature, flagging in a number of cases after a potentially contaminated component had been issued (see Table 6). Crucially, the primary culture of BCDP allowed for the interdiction of all Gram-negative BCDP contaminations over the period, as well as many other significant isolates, including *S. aureus* and Group C Streptococcus*. BCDPs yielded ‘significant’ pathogens more frequently than SDAP (14 vs. 10) despite being the minority component. This is a reminder that the contribution of four ‘donor BC’ to each pool leads to a greater likelihood of contamination (see Table 2). A modelling study, published after the introduction of our updated protocol, suggested two-step protocols were likely to perform better than single testing strategies for higher-risk contamination events [23]. However, as the authors comment, the probability of the scenarios they described occurring was unknown. Furthermore, they included contamination scenarios that were likely to demonstrate a difference in policy performance, the majority incorporating lag times of 48 h, which would not favour LVDS [23,24].

Two positive culture results, one confirmed (*S. epidermidis*) and one indeterminate/contaminant (*Bacillus* spp.) from 1468 expired pooled platelets tested (0.136%) over ten years, represented a crude estimate of the residual risk for the period prior to LVDS. As only a very small proportion of the outdates underwent screening, it was hypothesised that the combination of LVDS plus 12 h quarantine would result in a greater proportion of contaminants being identified and intercepted during live testing, thus yielding a safer product overall. There was also a clear opportunity for enhanced and more accurate microbiological surveillance by screening 100% of outdates with the new protocol.

### 4.3. Single-Donor Apheresis Platelets 2010–2020

For apheresis platelets, as one might expect, the combined positivity rates from either Day-1 (rate = 1:1535) or Day-4 (rate = 1:5962) testing were lower than that of BCDP (1:799; 1:2304, respectively) (chi-squared = 1150.07, 1 df, *p* < 0.00001). The latter was influenced by the contribution of four donors per pool, and the application of a more sensitive (later) test. Approximately 36% of SDAP components underwent a second Day-4 test to allow for expiry at Day-7. Significant isolates confirmed by the Day-4 test included *S. aureus* and a *Streptococcus infantarius;* the latter, arguably more of an issue for the donor than for a recipient, even if it had been infused. The vast majority of secondary cultures yielded low pathogenicity organisms.

The expired SDAP cultures also uncovered mostly low pathogenicity skin-flora-type organisms with the exception of one case of *Clostridium perfringens*,(indeterminate positive). *C. perfringens* has been implicated as a transfusion-relevant organism with fatal outcomes, which can affect more than one patient even from a single donation [9,25].

Low concentrations of bacteria obtained in the primary sample of SDAP collections taken as early as 12 h post-phlebotomy may explain the relatively low rate of culture positives in single-donor platelets (0.065%). The rate remained low even with the second Day-4 test at 0.017% (which must be interpreted with knowledge that the denominator represents tests rather than donations), whilst rising to 0.085% at expiry. The expired rate was derived from a very small sample of under 4000 tests over 10 years (three isolates, all indeterminate) but is indicative of missed contaminations in live testing. The newly employed (delayed) sample was expected to yield rates closer to a combination of the primary and secondary culture positive rate of the pre-2020 period. The SDAP contamination rates for 2021, 2022, and 2023 (until June) ranged between 0.01% and 0.124%. The CBS and NHSBT reported lower SDAP contamination rates than the IBTS with a ‘7-day expiry—single-test’ strategy (0.02 and 0.04%, respectively), despite collecting a higher proportional sample volume [14,15]. This aspect of testing is discussed below.

### 4.4. Limitations of IBTS LVDS Strategy for Apheresis Platelets (SDAP)

A notable weakness in the IBTS protocol is that the delayed sample is obtained from the ‘mother bag’ of the original collection, rather than each individual split, as described by McDonald et al. [14,26]. Previously, the second Day-4 test was applied to individual splits. The majority of single-donor apheresis donations are processed into double doses, and therefore a sample volume of 16 mL from the ‘mother bag’ in the new protocol represents 3.3% of double-dose donation and only 2.2% of a triple-dose collection (see Table S6). Upon review of ‘confirmed positive’ data relating to SDAP contaminations when all ‘splits’ relating to the original donation were available for testing, 73% (n = 31/42) were ‘*pan-contaminated*’. Of note, if the *indeterminate* cases where more than one split was available for testing are taken into account (culture of the available split did not confirm the presence of bacteria in the donation, n = 21), the rate of *pan-contamination* reduces to 49% (31/63). Kamel et al. demonstrated a minimal proportionalmother-bag sample volume of 3.8%, distributed across two bottles and obtained at 24–36 h, enhanced detection and interdiction of contaminated Day-5 SDAP. Therefore, it could be argued that an individual sample may not be absolutely necessary [27,28]. In addition, as commented by Benjamin and McDonald, haemovigilance data do not support a significant difference in STRs rates across various multiples of apheresis collections [8]. The IBTS will, in the near future, evaluate the possibility of testing each individual split from SDAP donations, the majority of which are double-dose donations (although 17% are triple dose). Alternatively, the approach of the CBS, which obtains a larger proportionate volume from the mother bag by utilising anaerobic bottles, could be considered, but we would have difficulty with the enhanced capacity required to incubate supplementary bottles [15]. Obtaining a test at an even later interval (e.g. no sooner than 48 h) for 7-day expiry [26] was not deemed operationally feasible for the IBTS.

### 4.5. Close Calls—Rationale for the Value of a 12 h Quarantine

In the consideration of added value and safety, it was estimated the 12 h hold (compared to the virtual absence of a procedure for quarantine previously) would facilitate the interdiction of the majority of significant contamination events, particularly those with fast- to moderately fast-growing organisms. Exceptions concerning the former would include *Staphylococcus* spp. and some of the *S. aureus* cases already mentioned.

Indeed, the case of *Streptococcus dysgalactiae*, which flagged at 9.36 h from a BCDP that was already infused, could have been avoided with a 12 h hold. Further discussion on this and other *S. dysgalactiae* cases can be found in Supplementary Materials, Section A5 [29]. The IBTS was also fortunate to intercept a single-dose SDAP donation confirmed to be contaminated with *Listeria monoctyogenes* prior to issue from an asymptomatic donor, which did not flag until 18 h post-incubation. Recently, in Italy, an unscreened PC led to a transfusion reaction with this species [30]. Vollmer et al. demonstrated 100% of bacteria in the pathogenic category were detected within 12 h, and the CBS reported with a pilot and real-time data that even a 6 h hold is robust in intercepting rapidly growing Gram-negative organisms such as *Klebsiella* spp. [15,31]. The IBTS time-to-detection (TTD) data (see Table 5, Table 7, Tables S1 and S2) supported the principle of the quarantine period, but a shorter quarantine may also be permissible in the context of targeting Gram negatives. We can see from Table 5, for example, that *Escherichia coli* and *Serratia marcesens* were detected within 6 and 8 h of primary culture, respectively. The quarantine period remains under review in the IBTS. The 12 h hold can hinder the supply of more urgently required platelet components. In these circumstances, IBTS physicians can order the nonstandard exceptional release of the component for a patient in need.

### 4.6. BCDP Results after Introduction of the Single-Test LVDS Protocol

Rates of bacterial detection in BCDP from the single-test protocol (0.191%) is higher than either of the pre-2020 Day-2 or Day-4 rates (0.125% and 0.043%), or a combination thereof (see Table 2 and Table 4, respectively) (chi-square = 10.6334, 1 df, *p* = 0.0011). This finding without the second Day-4 culture is interesting, as the timing and volume of the singular post-2020 sample was not altered and therefore was not expected to offer a change in sensitivity. A review of potential contributors to this observation in relation to component collection and processing did not reveal a likely explanation. (The * ChloraPrep product changed mid-study (2021); after an initial rise in the first 3 months, rates reverted to baseline). It could be that a much larger sample size is required to detect a true change in the rate of detection [23]. It has also been noted in the literature that there is a lack of controlled studies to establish the relative contribution of the variations in component collection and processing, in supporting/eliminating bacteria and/or influencing their detection [8]. The expired pooled rate, which is relatively high at 0.08%, is kept under monthly review and may be marginally representative of the now-absent second Day-4 culture. Outdates are said to be one of the best estimates of residual risk and the ’true probability of exposure’, but we do not measure other factors that are important in this regard, such as the average age of the PC when transfused, and the inoculum size at outdate [32]. In the context of clinical risk, it is likely that the platelet is issued and infused, prior to the inoculum reaching a level that would be detected at expiry.

### 4.7. Apheresis Platelet Contamination Rates Post-2020

For SDAP, the smaller representative sample volume acknowledged earlier as a potential weakness with the single test (secondary culture included a second Day-4 test on all available splits from single-, double-, or triple-dose SDAP donation) may have been offset by the effect of the delayed sample, as evidenced when comparing the rate post- and pre-LVDS 0.072% vs. 0.043% (Day-1 and Day-4 combined), respectively, (chi-square = 4.0687, 1 df, *p* = 0.0437).

Testing at outdate for SDAP post-2020 yielded only two positives over 5540 tests (rate 0.054%, confirmed (n = 2), indeterminate (n = 1)) compared to 0.085% at expiry pre-2020. The latter represents a small sample of less than 5% of the total number of apheresis collections over 10 years and should be interpreted with that in mind. We can see from the interdiction rate of over 70% post-2020 that the delayed sample allows for a larger proportion of contaminations to be captured with live in-date testing. It may be too early to tell if there has been a true change in the sensitivity of the updated SDAP test; direct comparisons are difficult to make knowing the second Day-4 culture was obtained from each dose previously, and the sample size since the changeover is relatively small. To fully realise the advantages of LVDS, it would appear that the IBTS may need to move towards individual split testing.

### 4.8. Bacterial Contamination and Detection Home and Away

LVDS has proved a successful measure in reducing STRs in several blood services such as the NHSBT and CBS, as previously mentioned [15]. Data from Héma-Québec is also supportive; after the implementation of LVDS in 2015, their detection rate of true positives increased from 0.013% to 0.019%; of note, the rate excludes *Cutibacterium acnes* [24]. It is unclear why the rates of contamination in Ireland are higher than countries that have adopted similar control measures (peak rate of 0.2% in 2021); there may be factors in the collection and/or processing which support bacterial growth and detection, or it may be that the initial contamination rate is simply higher and more pervasive from the outset. Nonetheless, the IBTS confirmed positive rates are within the boundaries of international norms (0.013–0.104%), and similar to those reported by Australia (confirmed and indeterminate rate of 0.22% for pools and 0.11% for apheresis) [8,33]. If the difference is true, the higher contamination rate in Ireland with mostly low pathogenicity bacteria has not translated into clinical harm—these rates are kept under close review. In relation to outdate data in SDAP, the IBTS falls within the boundaries of a number of prevalence estimates for post-primary culture bacterial contamination summarised by Kundrapu et al. [34]. They reported contamination rates ranging from 217 to 813 per million across several studies, which compares favourably to the IBTS pre- and post-LVDS *confirmed* outdate rates for all platelet types (201–441 per million, respectively). Noting the small sample sizes (n = 1 confirmed case pre-LVDS in BCDP, 0 in SDAP with 1–3% tested at expiry; n = 3 confirmed post-LVDS, 1 in BCDP, 2 in SDAP; 100% tested at expiry). These data are also suggestive of comparable effectiveness of the pre- and post-LVDS strategy.

### 4.9. Septic Transfusion Reactions

It has been estimated that the residual risk of bacterial safety could be as low as one in a million and therefore within reported estimates of virus-associated risk [18]. However, recent cases of STR in the United States are a stark reminder that even with culture independent proactive approaches to averting TABS, fatalities can still occur [35]. According to records from 2016–2022, the IBTS receives approximately one transfusion reaction report every week, annual range 67–79. The lack of a confirmed STR in Ireland may be influenced by the clinical characteristics of recipients, a significant proportion of whom would be receiving antimicrobials at the time of transfusion. National data indicates that 30–40% of patients in Irish hospitals are receiving antibiotics at any given time, thus providing a level of protection against STRs, even if high levels of antibiotic usage have distinct disadvantages and adverse consequences [36,37]. Another contributory factor may be the observation that an inoculum of 10^5^ CFU/mL is required for a clinical reaction to be apparent [3]. Although we have confidence in the haemovigilance process and each report is investigated individually with every effort to isolate a potential causative organism, we know that subclinical cases and even marked STRs could be unrecorded, or masked and obscured by illnesses with similar signs and symptoms. It has been estimated that passive surveillance can yield ten-fold lower STR rates than an active surveillance mechanism such that under reporting is an issue in this area [34,38,39].

Ireland has screened close to half a million platelet doses in less than two decades; it is inevitable that an STR will occur as reported by most of the larger blood establishments, irrespective of the risk mitigation strategy. The realistic goal of platelet screening is the interdiction of clinically significant levels of contamination, as sterility is not achievable. It is important that clinicians are aware not only of the ‘negative-to-date’ aspect of release criteria, but also of the limited sensitivity of the test applied compared to those used to screen for viral agents. False-negative screens have been estimated to occur in 10–50% of early sample screens [20]. Of course, TABS is not limited to platelet components, but an STR due to RBC-associated contamination has not been confirmed in Ireland; screening of BCDP offers a surrogate bacterial screen of red cell components, as demonstrated in Table 6. Co-contamination of RBC components was demonstrated in approximately 30% of cases, when the RBC culture yielded the same organism as the pool.

## 5. Limitations

The reporting of our data over the last 13 years was challenged by manually maintained databases and the complexity of the pre-November 2020 screening strategy. Several possible permutations for screening of a single donation could have occurred at this time, including primary, secondary, and expired cultures testing, as well as investigation of co-components after a primary or secondary positive culture. This was difficult to collate and report. We do not have visibility on the age of the platelet at the time of issue (nor transfusion in the hospital) to fully inform an assessment of risk post-changeover to the LVDS-type protocol. As described in the methods section, the definitions the IBTS apply to categorise data could be criticised. In particular, the ’indeterminate positive’ category includes cases that could have been true (confirmed) positive, for the want of the availability of a co-component that was already transfused (this is, of course, the nature of live testing) or if the concentration had reached the threshold for detection. The indeterminate *positive* category also includes *false* positives, as the organism detected in the culture may simply be a contaminant of the bottle and not the component itself. Direct comparisons (in the pre-2020 testing) of BCDP and SDAP donations are obscured by the SDAP rate for the Day-4 test being representative of the number of tests rather than the number of donations*. Limitations of the updated protocol have been highlighted in the text, e.g., mother-bag-only testing of apheresis donations and the loss of the second Day-4 screen for BCDP post-2020.

## 6. Concluding Remarks

The single test approach will continue to be used by the IBTS, as approximately 60% of contaminated components are being interdicted prior to transfusion; SDAP and BCDP are now both screened after an optimised interval post-collection, wastage has been reduced, and false flags appear to be decreasing. One could say that the system regularly falls short as contaminated platelet concentrates have been issued to patients and near misses have still occurred; however, our goal is to issue safe PCs and avoid morbidity and mortality. Zero risk is not achievable with any risk mitigation strategy, all of which have ‘failed’ at some point according to the literature. Although fatal septic transfusion reactions have not been reported in Ireland since implementation of BACT/ALERT 3D screening, transient bacteraemia leading to a mild or unnoticed reaction is more common than records will document. Perhaps the future will facilitate synthetically produced platelets in a high grade ‘pharmaceutical’ atmosphere, without the need for a living donor. More realistically, platelets could be cryopreserved or cold-stored rather than facilitating the propagation of bacteria at ‘room temperature’ prior to issue [40]. A basic intervention such as a donor arm ‘wash’ before the disinfection procedure might offer a simple additional bioburden reduction. For now, bacterial risk persists, and blood services will continue to upgrade their risk mitigation strategies to ensure platelet components are microbiologically safe.

## Figures and Tables

**Table 1:** Details of platelet component management.

		Primary Culture Time (h)	Volume (mL)	1st Culture Component(s) Sampled	2nd Culture Component(s) Sampled	Post-Sample Hold (h)	Length of Screening (d)
Pre-LVDS (2010–2020)	SDAP	12–24 (Day-1)	16 *	Pre-spilt (mother bag)	Each split	No formal quarantine	5 (7 **)
	BCDP	≥36 (Day-2)	16 *	Each pool	Each pool	No formal quarantine	5 (7 **)
Post-LVDS (2020-Present)	SDAP	≥36 (Day-2)	16 *	Pre-spilt (mother bag)	NA	≥12	7
	BCDP	≥36 (Day-2)	16 *	Each pool	NA	≥12	7

* 16 mL sample was split equally between aerobic and anaerobic bottles (8 mL into BPA; 8 mL into BPN); ** Expiry was extended from 5 to 7 days, if second culture performed on Day-5.

**Table 2:** Positivity rates of platelets screened pre-LVDS (January 2010 to November 2020).

Pre-LVDS 2010–2020	Total Platelets Screened	Total Positive Platelet Rate (%) 95% CI Incidence	Total SDAP Screened (Day-1)	Total SDAP Rate (%) 95% CI Incidence	Total SDAP Screened on Day-4 (Extended SDAP)	Total Extended SDAP Rate (%) 95% CI Incidence	Total BCDP Screened (Day-2)	Total BCDP Rate (%) 95% CI Incidence	Total BCDP Screened on Day-4 (Extended BCDP)	Total Extended BCDP Rate (%) 95% CI Incidence
Number of tests	287,698		110,497		89,426 *		64,732		23,043 *	
Confirmed	101	0.035 [0.029–0.043] (1:2848)	29	0.026 [0.018–0.038] (1:3810)	8 ^a^	0.009 [0.004–0.018] (1:11,178)	57	0.088 [0.067–0.114] (1:1136)	7	0.030 [0.012–0.063] (1:3292)
Indeterminate Positive	77	0.027 [0.021–0.033] (1:3736)	43	0.039 [0.028–0.052] (1:2570)	7	0.008 [0.003–0.016] (1:12,775)	24	0.037 [0.024–0.055] (1:2697)	3	0.013 [0.003–0.038] (1:7681)
Combined rate	178	0.062 [0.053–0.072] (1:1616)	72	0.065 [0.051–0.082] (1:1535)	15	0.017 [0.009–0.028] (1:5962)	81	0.125 [0.099–0.156] (1:799)	10	0.043 [0.021–0.080] (1:2304)
False Positive	202	0.070 [0.061–0.081] (1:1424)	95	0.086 [0.070–0.105] (1:1163)	29	0.032 [0.022–0.047] (1:3083)	67	0.104 [0.080–0.131] (1:966)	11	0.048 [0.024–0.085] (1:2095)

* Value represents all remaining platelets tested for expiry date extension on Day-4; ^a^ Includes two ’false negative’ SDAP that were negative at primary and secondary culture. Testing was performed upon visual inspection of aggregates in the platelets.

**Table 3:** Breakdown of testing per platelet type for all indeterminate positive results.

	Pre-LVDS		Post-LVDS	
	SDAP	BCDP	SDAP	BCDP
All components tested	20	9	3	3
Some components tested (not all available)	7	18	0	8
Components not available to test	23	0	3	0

**Table 4:** Positivity rates of platelets screened post-LVDS (November 2020 to June 2023).

Post-LVDS 2020–2023	Total Platelets Screened	Total Positive Platelet Rate (%) 95% CI Incidence	Total SDAP Screened (Day-2)	Total SDAP Rate (%) 95% CI Incidence	Total BCDP Screened (Day-2)	Total BCDP Rate (%) 95% CI Incidence
Number of tests	44,642		23,538		20,989	
Confirmed	40	0.090 [0.064–0.122] (1:1116)	11	0.047 [0.023–0.084] (1:2140)	29	0.138 [0.093–0.198] (1:724)
Indeterminate Positive	17	0.038 [0.022–0.061] (1:2626)	6	0.025 [0.009–0.055] (1:3923)	11	0.052 [0.026–0.094] (1:1908)
Combined rate	57	0.128 [0.97–0.165] (1:783)	17	0.072 [0.042–0.116] (1:1385)	40	0.191 [0.136–0.259] (1:525)
False Positive	3	0.007 [0.001–0.020] (1:14,881)	3	0.013 [0.003–0.037] (1:7846)	0	0.000 [0.000–0.018] (<1:20,989)

**Table 5:** Confirmed organisms pre-LVDS (January 2010 to November 2020).

Group	Organism	SDAP Primary Culture	BCDP Primary Culture	Mean TTD (h)	SDAP Extension Day-4	BCDP Extension Day-4	Infused N = 31 (%)
*Cutibacterium*	*Cutibacterium acnes/* *S. epidermidis*	-	1	93.1	-	-	0 (0)
	*Cutibacterium* *acnes*/spp.	21	29	92.51	1	3	26 (84)
CNS	*Coag Neg Staph*	2	1	46.96	1	-	1 (3)
	*S. epidermidis*	1	9	18.5	2	3	1 (3)
	*S. capitis*	2	2	28.92	-	-	0 (0)
	*S. saccharolyticus*	1	2	61.28	-	-	1 (3)
	*Microaerophilic* *Staphylococci*	-	1	69.4	-	-	0 (0)
Gram-positive cocci	*Kocuria varians*	-	1	59.52	-	-	0 (0)
Pathogenic Gram-positive rods	*Listeria monocytogenes*	1	-	18.5	-	-	0 (0)
*Staphylococcus aureus*	*S. aureus*	-	1	12.24	3	1	1 (3)
Streptococci and enterococci	*S. dysgalactiae*	-	5	8.74	-	-	1 (3)
	*S. mitis/oralis*	1	-	13.4	-	-	0 (0)
	*S. pneumoniae*	-	2	16.1	-	-	0 (0)
	*S. infantarius*	-	-	-	1	-	0 (0)
	*Peptostreptococcus micros*	-	1	55.9	-	-	0 (0)
Gram-negatives	*E. coli*	-	1	5.3	-	-	0 (0)
	*S. marcescens*	-	1	7.44	-	-	0 (0)

**Table 6:** Percentage of SDAP, BCDP, and associated products infused at time of recall.

		All Apheresis Splits Infused (%)	Associated Split(s) Infused (%)	BCDP-only Infused (%)	BCDP and Associated Red Cell Infused (%)	Associated Red Cell-only Infused (%)
Pre-LVDS	Apheresis (n = 87)	24 (28)	13 (15)	-	-	-
	BCDP (n = 91)	-	-	37 (41)	2 (2)	4 (4)
Post-LVDS	Apheresis (n = 17)	3 (18)	1 (6)	-	-	-
	BCP (n = 40)	-	-	10 (25)	8 (20)	3 (8)

**Table 7:** Confirmed organisms post-LVDS (November 2020 to June 2023).

Group	Organism	SDAP Primary Culture	Expired SDAP	BCDP Primary Culture	Expired BCDP	Mean TTD (h)	Infused (%)
Skin commensals	*C. acnes*	5	1	17	1	98.86	13 (93)
’Coagulase-negative staphylococci‘ *	*S. capitis*	1	-	0	-	19.92	0 (0)
	*S. epidermidis*	1	-	1	-	19.32	0 (0)
	*S. lugdunensis*	-	-	1	-	19.44	0 (0)
	*S. saccharolyticus*	-	-	7	-	50.98	1 (7)
	Mixed skin flora	-	-	1	-	21.12	0 (0)
*S. aureus*	*S. aureus*	2	1	1	-	8.64	0 (0)
Streptococci and Enterococci	*S. gallolyticus*	1	-	-	-	9.36	0 (0)
	*S. dysgalactiae*	-	-	1	-	9.12	0 (0)
	*E. casseliflavus*	1	-	-	-	4.08	0 (0)

* Organism identification by reference laboratory.

**Table 8:** Positivity rates of expired platelets screened post-LVDS (November 2020 to June 2023).

Expired Platelets Post-LVDS	Total Expired Platelets	Total Expired Platelets Rate (%) 95% CI Incidence	Expired SDAP	Expired SDAP Rate (%) 95% CI Incidence	Expired BCDP	Expired BCDP Rate (%) 95% CI Incidence
Number of Tests	6809		5540		1269	
Confirmed	3	0.044	2	0.036	1	0.079
		[0.009–0.129]		[0.004–0.130]		[0.002–0.438]
		(1:2270)		(1:2770)		(1:1269)
Indeterminate Positive	1	0.015	1	0.018	0	0.000
		[0.000–0.082]		[0.000–0.101]		[0.000–0.290]
		(1:6809)		(1:5540)		(<1:1269)
Combined rate	4	0.059	3	0.054	1	0.079
		[0.016–0.150]		[0.011–0.158]		[0.002–0.438]
		(1:1723)		(1:1847)		(1:1269)
False Positive	3	0.044	3	0.054	0	0.000
		[0.009–0.129]		[0.011–0.158]		[0.000–0.290]
		(1:2270)		(1:1847)		(<1:1269)

**Table 9:** Positivity rates of expired platelets screened pre-LVDS (2010 to November 2020).

Expired Platelets Pre-LVDS	Total Expired Platelets	Total Expired Platelets Rate (%) 95% CI Incidence	Expired SDAP	Expired SDAP Rate (%) 95% CI Incidence	Expired BCDP	Expired BCDP Rate (%) 95% CI Incidence
Number of Tests	4987		3519		1468	
Confirmed	1	0.020 [0.001–0.112] (1:4987)	0	0.000 [0.000–0.105] (<1:3519)	1	0.068 [0.002–0.379] (1:1468)
Indeterminate Positive	4	0.080 [0.022–0.205] (1:1247)	3	0.085 [0.018–0.249] (1:1173)	1	0.068 [0.002–0.379] (1:1468)
Combined rate	5	0.100 [0.033–0.234] (1:997)	3	0.085 [0.018–0.249] (1:1173)	2	0.136 [0.017–0.491] (1:734)
False Positive	17	0.341 [0.199–0.545] (1:293)	12	0.341 [0.176–0.595] (1:293)	5	0.341 [0.111–0.793] (1:294)

## Data Availability

The data presented in this study are available on request from the corresponding author.

## Supplementary Materials

The following supporting information can be downloaded at: https://www.mdpi.com/article/10.3390/microorganisms11112765/s1, Table S1: Organisms detected in indeterminate investigations pre-LVDS; Table S2: Indeterminate positive organisms post-LVDS (November 2020–June 2023); Table S3: Significant organisms from confirmed investigations; Table S4: Significant organisms from indeterminate investigations; Table S5: Organisms isolated from positive expired platelets, pre- and post-LVDS; Table S6: Percentage of SDAP platelets collected in single (SD), double (DD), and triple (TD) doses; Section A: Additional information on IBTS operations. Refs. [41,42,43,44,45,46] are cited in the supplementary materials.
