# Supplementary material for: Changing Strategies for the Detection of Bacteria in Platelet Components in Ireland: From Primary and Secondary Culture (2010–2020) to Large Volume Delayed Sampling (2020–2023)
Source: Microorganisms. 2023 Nov 14;11(11):2765. doi: 10.3390/microorganisms11112765 (PMC10673373; doi:10.3390/microorganisms11112765)
Supplement: Supplementary file 1 [file microorganisms-11-02765-s001.zip › microorganisms-2669094-supplementary.pdf]

microorganisms

Article

Changing Strategies for the Detection of Bacteria in Platelet components in Ireland: from Primary and Secondary culture (2010–2020) to Large Volume Delayed Sampling (2020–2023)

Niamh O'Flaherty *, Louise Bryce, James Nolan and Mark Lambert

Irish Blood Transfusion Service, National Blood Centre, Ireland; louise.bryce@ibts.ie (L.B.); mark.lambert@ibts.ie (M.L.)
* Correspondence: niamh.oflaherty@ibts.ie

Supplementary Materials

Table S1. Organisms detected in indeterminate investigations pre-LVDS.

Group	Organism	SDAP Primary Culture	BCDP Primary Culture	Mean TTD (h)	SDAP Extension Day-4	BCDP Extension Day-4	Infused N=45 (%)
Cutibacterium	*Propionibacterium granulosum*	0	1	74.2	-	-	0 (0)
	GPB (*Cutibacterium* spp.)	1	-	114.5	-	-	1 (2)
	Cutibacterium acnes/spp.	16	7		1	0	23 (51)
'Coagulase Negative stapyhlococci'*	*Coag Neg Staphylococcus* spp.	4	1	61.77	-	1	3 (7)
	S. capitis	3	2	31.58	-	-	0 (0)
	S. epidermidis	5	1	24.6	2	-	2 (4)
	S. hominis	-	-	-	-	1	0 (0)
	S. hominis & S. lugdunensis	-	1	17.0	-	-	0 (0)
	S. saccharolyticus	1	2	61.8	-	-	3 (7)
	S. warneri	-	-	-	2	-	0 (0)
	S. auricularis	-	-	-	-	1	0 (0)
Corynebacterium	*Corynebacterium* spp.	3	3	89.4	-	-	5 (11)
Streptococci	*S. pneumoniae*	1	-	18.7	-	-	1 (2)
	Microaerophilic Streptococci	-	-	-	-	1	1 (2)
Bacillus and other spore formers	*Paenibacilus lautus*	-	1	40.3	-	-	0 (0)
	AnO_2 Gram Positive Bacilli	1		117.1	-	-	1 (2)
	Bacillus cereus	1	-	12.0	-	-	0 (0)
	Bacillus licheniformis	1	-	27.4	-	-	0 (0)
	Bacillus spp.	4	1	27.5		-	1 (2)
	Bacillus subtilis	-	1	16.6	-	-	0 (0)
Gram Negatives	*Desulfovibrio* spp.	-	1	50.6	-	-	1 (2)
	Bacteroides vulgatu/ Colinsella aerofacians	1	-	30.5	-	-	0 (0)
	Acinetobacter spp., *Bacillus* spp., *Peptostreptoccus*	1	-	16.1	-	-	1 (2)
Others	*Collinsella aerofaciens*	-	1	53.8	-	-	1 (2)
	Kocuria varians	-	1	67.9	-	-	1 (2)
	Mixed growth: S.capitis & CNS	-	-	-	1	-	0 (0)

* Organism identification from the reference laboratory.

Table S2. Indeterminate positive organisms isolated post-LVDS (November 2020 – June 2023).

Group	Organism	SDAP Primary Culture	Expired SDAP	BCDP Primary Culture	Expired BCDP	Mean TTD (h)	Infused (%)
Skin commensals	*C. acnes*	4	1	6	-	103.6	9 (82)
'Coagulase Negative staphylococci'*	*S. capitis*	-	-	1	-	43.44	0 (0)
	S. hominis	1	-	-	-	21.6	0 (0)
	S. epidermidis	-	-	1	-	14.64	1 (9)
	S. saccharolyticus	-	-	2	-	102.6	1 (9)
Streptococci	*S. mitis/oralis*	-	-	1	-	23.76	0 (0)
Mixed culture	*Neisseria mucosa & S. mitis/oralis*	1	-	-	-	0.22	0 (0)

* Organism identification by reference laboratory.

Table S3. Significant organisms from confirmed investigations.

Group	Significant Organisms	SDAP n=10	BCDP n=14	Mean TTD	Infused N	Comments
Gram Negatives	*E. coli*	-	1	5.28	0	
	S. marcescens	-	1	7.44	0	
Pathogenic Gram-Positive rods	*Listeria monocytogenes*	1	-	18.48	0	
Staphylococcus aureus	*S. aureus*	3[a]	3[b]	9.54	-	-
	False Negatives	3	-	-	1	Initial screening test of all 3 'False Negative' apheresis platelets tested negative. The platelets were tested again when aggregates were observed by visual inspection
	S. lugdunensis	-	1	19.44	0	
Streptococci and enterococci	*S. dysgalactiae*	-	6	8.8	1	Pool infused. One associated red cell tested positive for *S. dysgalactiae*
	S. infantarius	1	-	-	0	Apheresis platelet tested after extension on Day-4
	S. pneumoniae	-	2	16.08	0	
	S. gallolyticus	1	-	0.39	0	
	E. casseliflavus	1		0.17	0	

[a] Includes one SDAP tested on Day 4; [b] Includes 1 BCDP tested on Day 4

Table S4. Significant organisms from indeterminate investigations.

Group	Significant Organisms	Number of Isolates	Mean TTD	Infused N	Comments
Bacillus spp. and other spore formers	*Bacillus cereus*	1	12	-	
	Bacillus spp.	2	27.5	1	Pool infused. All associated products tested negative
	Clostridium perfringens	1	-	-	Expired SDAP
Streptococci	*Microaerophilic Streptococci*	1	-	1	Pool infused. All associated products tested negative
	S. mitis/oralis	1	23.76	-	
	S. pneumoniae	1	18.72	1	Both splits of SDAP infused
CNS	*S. hominis & S. lugdunensis*	1	17.04	-	
Others	*Acinetobacter spp., Bacillus spp., Peptostreptococcus anaerobius*	1	16.08	1	Split 1 infused. Split 2 tested negative
	Bacteroides vulgatu/Colinsella aerofacians	1	30.48	-	

Table S5. Organisms isolated from positive expired platelets, pre- and post-LVDS.

Time Period	Organism	Expired SDAP	Expired BCDP	Category	Infused	Follow-up testing
**Pre-LV DS**	*Clostridium perfringens*	1		Indeterminate	No	Both splits negative
	Corynebacterium spp	1		Indeterminate	Split 1	Split 2 negative
	Corynebacterium jeikeium	1		Indeterminate	Split 1	Splits 2 and 3 negative
	Bacillus circulans		1	Indeterminate	No	Pool and 4x RCC negative
	S. epidermidis		1	Confirmed	No	Pool repeat test positive, 4x RCC negative
**Post-LV DS**	*C. acnes*	1[a]	1[b]	Confirmed	No	-
	C. acnes	1		Indeterminate	No	Splits 1 and 2 both negative
	S. aureus	1		Confirmed	No	Primary culture false negative; both splits tested positive for *S. aureus* following observation of aggregates in both splits

[a]Split 2 positive for *C. acnes.* [b]Pool tested negative, 1x RCC tested positive for *C. acnes*

Table S6. Percentage of SDAP platelets collected in single (SD), double (DD), and triple (TD) doses; and % volume of PC cultured.

Year	PC Type	Total	SDAP Type	SD	% of Total	Mean Volume	% Volume Cultured*
2022	SDAP	5,535	SD	284	5.1%	260	6.2%
			DD	4,291	77.5%	244	6.6%
			TD	960	17.3%	239	6.7%
	BCDP	2,002	-	-	-	333	4.8%
2021	SDAP	6,320	SD	357	5.6%	243	3.3%
			DD	5,145	81.4%	242	3.3%
			TD	818	12.9%	237	3.4%
	BCDP	1,988	-	-	-	328	4.9%
2020	SDAP	6,382	SD	559	8.8%	237	2.3%
			DD	5,226	81.9%	243	2.2%
			TD	597	9.4%	237	2.3%
	BCDP	1,832	-	-	-	324	4.9%

*16 ml inoculated in BPA and BPN bottles (8 ml each);); '% volume cultured' is the percentage of average volume that 16ml represents, e.g. 2020 BCDP; 16ml of 324ml = 4.9%.

Section A (1-7)

A1. Leucodepletion

All products produced by the IBTS are leucodepleted; red cells are leucodepleted by integrated filters on closed-system blood collection bags (LQT614B and FQE614B blood packs; MacoPharma, France); BCDP are leucodepleted by integrated filters on TACSI PL sets; SDAP are leucodpeleted by apheresis processing. Leucodepletion is confirmed by flow cytometery.

A2. Bioburden Reduction

The IBTS employs additional measures to reduce the likelihood of donor-derived bacteria contaminating blood products. These measures include scheduled clinic cleaning, donor health assessment, donor arm disinfection with ChloraPrep™ (Becton Dickinson, New Jersey, US), phlebotomist training, and technique proficiency assessment, diversion of first 35 ml into sample pouch, use of closed blood bag system, and use of sterile docking device for any secondary processing.

At the IBTS donor arms are disinfected through the application of ChloraPrep (2% chlorhexidine with 70% isopropyl alcohol). Briefly, ChloraPrep™ 1 ml applicator is applied to the *antecubital fossa*, 6 cms above and below, and 5 cms left to right of the venepuncture site for 30 seconds; 30 seconds drying time must elapse prior to venepuncture.

A3. Donor Follow-Up Associated with Positive BacT/ALERT Bottle

Following identification of organisms in platelet products, all positive cultures are reviewed by the medical team and consultant microbiologist in real-time to assess for the need to contact the donor (when identified) to assess their health status. This was initiated for all of the organisms in the 'significant confirmed' category (e.g., *Steptococcus infantarius, Steptococcus gallolyticus, Enterococcus casseflavus, Serratia marcesens*). Donors are swabbed in the *antecubital fossa* and anterior nares. Swabs are investigated and identification performed by the same medical microbiology laboratory. If the same organism is identified by this laboratory as that in the component, then the donor is permanently retired from donating.

A4. Patient Treatment when Transfused with Platelets with Positive BacT/ALERT Bottle

Patients who have received platelets from a potentially contaminated SDAP or BCDP are investigated for potential TTI, on advice of the IBTS medical team. Patients are treated according to local hospital policy; IBTS would suggest blood cultures in the case of a significant organism. Advice can be sought from the clinical microbiologist at the IBTS in this regard.

A5. Cases of interest (see also *Discussion)*

i. ***Streptococcus dysgalactiae***: Fortunately, the immune-compromised recipient with acute myeloid leukaemia was already on a penicillin-based antimicrobial for febrile neutropenia at the time of infusion, and did not manifest any acute reaction to the transfused component. Six months later, a donor who contributed to this pool was linked to another BCDP culture, which flagged at the exact same time interval, again with *Streptococcus dysgalactiae*. The component was intercepted on this occasion, albeit after it was issued to a paediatric hospital. The healthy donor, common to both events, was permanently deferred without any further molecular or confirmatory investigations, as there was adequate circumstantial evidence that the cases were related. A similar event was reported in Japan (2018) where STRs occurred 6 months apart after unscreened PCs, which grew genetically similar Group G Streptococci (GGS) were linked to a common regular donor. They reported GGS as the causative agent in 24% of TABS cases over a ten year period [1].

ii. ***Staphylococcus epidermidis***: An Irish recipient of a BCDP contaminated with *Staphylococcus epidermidis* transfused during prosthetic valve insertion surgery received a six-week course of antimicrobials to avoid any potential for seeding of the newly inserted valve. Although blood cultures were repeatedly negative and the antibiotic course was precautionary, the detection of the organism, albeit at a stage later than desirable, was beneficial for the recipient who may otherwise have developed a biofilm-related *S. epidermidis* endocarditis. For all of these reasons, skin-flora-type contaminants cannot be dismissed, and follow-up of recipients is carefully managed by the IBTS medical team and their clinician colleagues in the hospitals.

iii. ***Serratia marcescens***: A 'close call' with a *Serratia marcesens* contaminated pool may have led to a very different outcome had the unquarantined unit been issued. The contamination of the associated plasma allowed for the identification of a reportedly well male with haemochromatosis, presenting as a blood donor. As the organism was also identified in the donor's recovered plasma and therefore less likely to have been from an environmental or skin source, it was hypothesised that symptoms of hyperferritinaemia may have masked a transient bacteraemia. This case was closely investigated and was ultimately felt to have been a donor-derived event. An unresolved aspect of the case was the detection of a genetically related *S. marcesens* isolate from a swab of a platelet pack resting on an agitator a number of weeks after the contamination occurred. This finding was reminiscent of the nonsterile blood packs which had to be withdrawn by the manufacturer after causing S. marcesens related STRs (some fatal) in the 1990s in both Denmark and Sweden [2,3]. No such issue was reported or confirmed by the manufacturer on this occasion. Hence, as a response to the threat of releasing a component prior to automated detection, a 12-hour quarantine for all PC was introduced, which is still in place today.

***A6.* Macroscopic evidence of platelet contamination and Staphylococcus aureus**

The visual inspection by blood bank staff of PC for gross signs of contamination such as aggregates and lack of swirling, remains a critical line of defence against recipient harm [4]. At the IBTS, this crude but cost-efficient method of bacterial surveillance inter-

cepted the delivery to hospitals of two out of six of seven *S. aureus*-contaminated SDAP collections splits (in both the pre- and post-LVDS period). In the false negative *S. aureus* SDAP cases, the culture was positive in both splits before being issued in one case (2020). Whilst in another, one split of two had been infused (without a reaction in the patient who was receiving antimicrobials), before the second split was noted to have aggregates in the hospital. This later case highlights the critical importance of timely communication to the blood establishment of abnormalities observed in blood component appearance; especially if the infusion of co-components can be averted. This is an action that could be lifesaving.

In the UK, an *S. aureus*-contaminated component with a false-negative screen led to an STR and morbidity, whilst three other cases were averted due to the vigilance of staff members [5]. *Staphylococcus aureus* has particular characteristics which make it a 'triple threat' of the Gram-positive transfusion-relevant organisms; it can form biofilms, escape routine detection by culture, and cause significant transfusion reactions due to super antigen and enterotoxin production. These effects appear to be enhanced in platelet storage compared to standard culture conditions [4,6].

Donors linked to these cases in Ireland are swabbed in the antecubital fossa and anterior nares, and if the same biotype is identified by the reference laboratory as that in the component, they are permanently retired from donating.

A7. Frequently observed contaminants

International literature and our local experience would support the understanding that *Cutibacterium* spp. (previously *Propionibacterium)* and Coagulase-negative Staphylococci are not usually associated with morbidity in the recipient when infused. Furthermore, a criticism of the 'negative-to-date' strategy and component monitoring after issue, is the 'retrospective' nature of the process, which adds to the workload of healthcare professionals, and patient related distress, especially when results are inconsequential and ultimately may not be confirmed after the notification [7]. This pattern is not abating as *Cutibacterium* appears to be accounting for an even greater proportion of positive detections post-introduction of LVDS in late 2020 (126/351, 36% pre changeover vs. 35/61, 57% after, see tables 2 and 4), and a higher rate of anaerobic bottle flags (along *with S. saccharolyticus*). The reasons for this are unclear. Although researchers did not confirm the existence of a 'common microbiome' in the examination of healthy blood cultures; *Cutibacterium acnes* was the most commonly detected organism in 4.7% of the population they studied [8]. Overall, the precautionary approach must be upheld and STR including deaths due to CNS-contaminated units have been reported after transfusion and in clinical practice. *C. acnes* is well documented as an agent of prosthetic (joint, cardiac valve) indwelling device infections, even if rarely reported as an agent of TTI [7]. *Staphylococcus saccharolyticus*, a strict anaerobe with a lengthy 50-hour average TTD has repeatedly been detected in IBTS pools since 2020; this may be a feature of the identification system used by the reference laboratory rather than any systematic reason for contamination. Both *Cutibacterium acnes* and *Staphylococcus saccharolyticus* have been shown to have the ability not only to grow as biofilms but also to adhere to the interior of platelet packs [9].

References

1. Kozakai, M.; Matsumoto, M.; Takakura, A.; Furuta, R.A.; Matsubayashi, K.; Goto, N.; Satake, M. Two cases of *Streptococcus dysgalactiae* subspecies equisimilis infection transmitted through transfusion of platelet concentrate derived from separate blood donations by the same donor. *Vox Sang.* **2023**, *118*, 582–586.
2. Heltberg, O.; Skov, F.; Gerner-Smidt, P.; Kolmos, H.J.; Dybkjaer, E.; Gutschik, E.; Jerne, D.; Jepsen, O.; Weischer, M.; Frederiksen, W.; et al. Nosocomial epidemic of *Serratia marcescens* septicemia ascribed to contaminated blood transfusion bags. *Transfusion* **1993**, *33*, 221–227.
3. Hogman, C.F.; Fritz, H.; Sandberg, L. Posttransfusion *Serratia marcescens* septicemia. *Transfusion* **1993**, *33*, 189–191.

4. Loza-Correa, M.; Kou, Y.; Taha, M.; Kalab, M.; Ronholm, J.; Schlievert, P.M.; Cahill, M.P.; Skeate, R.; Cserti-Gazdewich, C.; Ramirez-Arcos, S. Septic transfusion case caused by a platelet pool with visible clotting due to contamination with *Staphylococcus aureus*. *Transfusion* **2017**, *57*, 1299–1303.
5. McDonald, C.; Allen, J.; Brailsford, S.; Roy, A.; Ball, J.; Moule, R.; Vasconcelos, M.; Morrison, R.; Pitt, T. Bacterial screening of platelet components by National Health Service Blood and Transplant, an effective risk reduction measure. *Transfusion* **2017**, *57*, 1122–1131.
6. Chi, S.I.; Yousuf, B.; Paredes, C.; Bearne, J.; McDonald, C.; Ramirez-Arcos, S. Proof of concept for detection of staphylococcal enterotoxins in platelet concentrates as a novel safety mitigation strategy. *Vox Sang.* **2023**, *118*, 543–550.
7. Thyer, J.; Perkowska-Guse, Z.; Ismay, S.L.; Keller, A.J.; Chan, H.T.; Dennington, P.M.; Bell, B.; Kotsiou, G.; Pink, J.M. Bacterial testing of platelets—Has it prevented transfusion-transmitted bacterial infections in Australia? *Vox Sang.* **2018**, *113*, 13–20.
8. Tan, C.C.S.; Ko, K.K.K.; Chen, H.; Liu, J.; Loh, M.; Chia, M.; Nagarajan, N.; SG10K_Health Consortium. No evidence for a common blood microbiome based on a population study of 9,770 healthy humans. *Nat. Microbiol.* **2023**, *8*, 973–985.
9. Kumaran, D.; Kalab, M.; Rood, I.G.H.; de Korte, D.; Ramirez-Arcos, S. Adhesion of anaerobic bacteria to platelet containers. *Vox Sang.* **2014**, *107*, 188–191.
